# Integrated Genomic and Greenhouse Assessment of a Novel Plant Growth-Promoting Rhizobacterium for Tomato Plant

**DOI:** 10.3389/fpls.2021.660620

**Published:** 2021-03-30

**Authors:** Maria Chiara Guerrieri, Andrea Fiorini, Elisabetta Fanfoni, Vincenzo Tabaglio, Pier Sandro Cocconcelli, Marco Trevisan, Edoardo Puglisi

**Affiliations:** ^1^Department for Sustainable Food Process, Università Cattolica del Sacro Cuore, Piacenza, Italy; ^2^Department of Sustainable Crop Production, Università Cattolica del Sacro Cuore, Piacenza, Italy

**Keywords:** *Klebsiella variicola*, *Azospirillum brasilense*, PGPR, genome analyses, inoculation

## Abstract

Plant growth promoting rhizobacteria (PGPR) can display several plant-beneficial properties, including support to plant nutrition, regulation of plant growth, and biocontrol of pests. Mechanisms behind these effects are directly related to the presence and expression of specific genes, and different PGPR strains can be differentiated by the presence of different genes. In this study we reported a comprehensive evaluation of a novel PGPR *Klebsiella variicola* UC4115 from the field to the lab, and from the lab to the plant. The isolate from tomato field was screened *in-vitro* for different activities related to plant nutrition and growth regulation as well as for antifungal traits. We performed a functional annotation of genes contributing to plant-beneficial functions previously tested *in-vitro*. Furthermore, the *in-vitro* characterization, the whole genome sequencing and annotation of *K. variicola* UC4115, were compared with the well-known PGPR *Azospirillum brasilense* strain Sp7. This novel comparative analysis revealed different accumulation of plant-beneficial functions contributing genes, and the presence of different genes that accomplished the same functions. Greenhouse assays on tomato seedlings from BBCH 11–12 to BBCH > 14 were performed under either organic or conventional management. In each of them, three PGPR inoculations (control, *K. variicola* UC4115, *A. brasilense* Sp7) were applied at either seed-, root-, and seed plus root level. Results confirmed the PGP potential of *K. variicola* UC4115; in particular, its high value potential as indole-3-acetic acid producer was observed in increasing of root length density and diameter class length parameters. While, in general, *A. brasilense* Sp7 had a greater effect on biomass, probably due to its high ability as nitrogen-fixing bacteria. For *K. variicola* UC4115, the most consistent data were noticed under organic management, with application at seed level. While, *A. brasilense* Sp7 showed the greatest performance under conventional management. Our data highlight the necessity to tailor the selected PGPR, with the mode of inoculation and the crop-soil combination.

## Introduction

Plant growth promoting rhizobacteria (PGPR) are a heterogenous group of soil-dwelling bacteria able to efficiently colonize plants root system, enhancing plant nutrition, stress tolerance and health (Vacheron et al., 2013). PGPR can improve crops fitness either directly and indirectly. Direct processes include the promotion of alternative nutrient uptake pathway, the solubilization or fixation of chemical nutrients, and the production of various phytohormones; on the other hand indirect processes reduce or prevent the deleterious effects of one or more phytopathogenic organisms, through the synthesis of numerous antimicrobial compounds, and/or inducing systemic resistance (Oleńska et al., 2020). Since the recognition of their capabilities, several hundreds of candidate PGPR strains have been screened and evaluated in laboratory, greenhouse and field studies across the world (Martínez-Viveros et al., 2010). However, despite extensive literature on the mode of actions of PGPR, the implementation of this biotechnology in the agriculture industry, has been limited due to the variability information of the effectiveness of PGPR on plant growth, which could influence crop production (Vejan et al., 2016).

In recent years, the genome characterization of emblematic PGPR model strains have uncovered the molecular basis of some of their beneficial effects, leading the identification of genes that are involved in the PGPR-plant cooperation (Bruto et al., 2014; Liu et al., 2016; Shariati et al., 2017). According to these studies, many PGPR strains are multifunctional, i.e., they harbor more than one plant-beneficial properties, and this suggests the presence of conservative genes commonly distributed among different genera of microorganisms. However, a recent study based on ancestral character reconstruction, has proposed that PGPR-plant cooperation may have established separately in various taxa, yielding PGPR strains that use different gene assortments (Bruto et al., 2014). A holistic comparative discussion between *in-vitro* and *in-vivo* assay, and genomics could enhance the observation how these different assortments of plant-beneficial functions contributing genes (PBFC genes) affect the eukaryotic host.

A concrete observation about contribution of different multifunctional PGPR, can be performed using greenhouse horticultural crop, since different promotion capacity can be more easily determined under semi-controlled conditions (Ruzzi and Aroca, 2015). So far, organic production of high-value horticulture is one of the most important areas of PGPR application (Olowe et al., 2020). Tomato (*Solanum lycopersicum* L.) is one of the most demanding high-value horticultural crops worldwide, after potato (Singh et al., 2018), and is a good crop model study for PGPR effectiveness, because in the majority of cases, inoculation with PGPR always determined an increase in plant root length and plant productivity (Ruzzi and Aroca, 2015).

The genus *Klebsiella*, is one of the common genera detected in the rhizosphere system (Podile and Kishore, 2006), and the species *Klebsiella variicola* strains have been found to naturally colonize the rhizosphere of tomato plant (Guerrieri et al., 2020; Sunera et al., 2020). In particular, the plant growth-promoting (PGP) capacity of distinct strains of *K. variicola* were confirmed in glasshouse experiments as well as in field trials, both in terms of yield increase and product quality improvement (Kim et al., 2017; Yang and Yang, 2020). Plants seem to be the natural niches for this species, and its dominant feature is the capability to fix N_2_ (Chen et al., 2016). Actually, *Klebsiella sp*. are among the major free-living nitrogen fixing bacteria, together with *Azospirillum brasilense, Pantoea agglomerans, Burkholderia sp., Serratia sp*. (Bhattacharjee et al., 2008). Moreover, different PGP traits such as phosphorus solubilization, indole-3-acetic acid (IAA) production, and biocontrol activity were identified in distinct strains of *K. variicola* (Liu et al., 2014; Sekhar and Thomas, 2015). Despite the high PGP potential of this species, *K. variicola* is also known for its potential as opportunistic pathogens, with a number of strains of this species known to cause nosocomial respiratory and urinary tract infection, and pediatric outbreaks (Barrios-Camacho et al., 2019). A few *K. variicola* genomes analysis are currently reported, but the majority of investigations have focused on their pathogenic genes (Srinivasan and Rajamohan, 2020). By contrast, a shortage of studies is referred to the genes that contribute to the beneficial PGP activity of this species, except for nitrogen fixation.

Here, we used the high plant growth promoting potential of *K. variicola* strain UC4115 as a case study to present a comprehensive overview of an evaluating approach of PGPR: from the plant to the lab, through the phenotypic and genomic characterization of the isolate, and from the lab to the plant, through the direct observation of PGPR-plant beneficial cooperation. To obtain a better understanding of the effects of different PGPR on plant growth, the genomic and agronomical evaluation of *K. variicola* UC4115 was compared with that of *Azospirillum brasilense* Sp7, one of the best studied PGPR at present (Cassán and Diaz-Zorita, 2016). *K. variicola* UC4115 was isolated from the rhizosphere of tomato plant, after a long-term reduce tillage soil management, based on its nitrogen-fixation ability. Strains *K. variicola* UC4115 and *A. brasilense* Sp7 were screened *in vitro* for PGP activities related to (i) nutrition, including phosphate (P) solubilization and siderophores synthesis, (ii) the production of growth hormones like IAA, and (iii) antagonism against fungal pathogen such as *Sclerotinia sclerotiorum*. Later, whole genome sequencing and annotation was performed to identify target genes that contribute to the beneficial interaction between bacteria and plants. Finally, an *in vivo*-greenhouse experiment on tomato seedlings from BBCH (Biologische Bundesanstalt, Bundessortenamt, and CHemical industry) 11–12 to BBCH > 14 was performed in both organic and conventional management, accomplished with three inoculations (control, *K. variicola* UC4115, *A. brasilense* Sp7) at seed, root and seed and root level.

## Materials and Methods

### Isolation of PGPR From Rhizosphere Soil and Cultural Conditions

PGP rhizobacteria strain UC4115 was isolated from the rhizosphere of *Solanum lycopersicum* L., cultivated with conservation agriculture practices (i.e., reduced tillage and cover crops), in a commercial field situated in Gabbioneta-Binanuova (45°12'03.0” N; 10°12'27.8” E), Cremona, Po Valley (Northern Italy), following the method described by Guerrieri et al. (2020). Briefly, to isolate the rhizosphere bacteria, root samples were shaken vigorously to remove not tightly adhering soil. The root system was washed with sterile physiological water added with Tween 80 (0.01% v/v), and the mixture was incubated at 25°C for 90 min with shaking at 180 rpm. The resulting suspensions were serially diluted (10^−3^) and 0.1 mL aliquots were used to grow cultures in N-free semi-solid media, NFb (New Fabio Pedrosa), and incubated 4–7 d at 30°C until the growth of a veil-like pellicle near the surface of the culture medium, and the color change of the medium from green-blueish to blue. Subculturing the isolate three times on LB agar plates (Luria-Bertani) (Oxoid, Basingstoke, UK) resulted in pure colonies of rhizosphere bacteria.

*Azospirillum brasilense* Sp7 (DSMZ 1690) was provided by the German Type Culture Collection (DSMZ, Braunschweig, Germany). Bacterial strain was inoculated in LB Broth and incubated at 30°C for 48 h, with shaking at 180 rpm.

### Taxonomic Identification of Bacterial Isolate UC4115

According to Guerrieri et al. (2020), the extracted DNA was amplified with 16S rRNA, using the universal primers P1 (5′-GCGGCGTGCCTAATACATGC-3′) and P6 (5′-CTACGGCTACCTTGTTACGA-3′) (Di Cello and Fani, 1996). Sanger sequencing of PCR products was carried out at GATC Biotech (Ebersberg, Germany). The obtained 16S ribosomal DNA sequences were compared with others in the GenBank database, through the NCBI-BLAST server, at https://blast.ncbi.nlm.nih.gov/Blast.cgi.

### *In vitro* Assessment of PGP Traits

The PGP traits of *K. variicola* UC4115 and *A. brasilense* Sp7 were assessed following the methods described by Guerrieri et al. (2020). Briefly, for qualitative estimation of tri-calcium phosphate solubilization, the strains were spot inoculated on GY/Tricalcium phosphate medium (Ambrosini and Passaglia, 2017), and incubated at 30°C. After 7 days of incubation plates were observed for development of a clear halo zone around the colony, the halo's diameter was evaluated according to Ambrosini and Passaglia (2017). The phytohormone IAA production was estimated using the Salkowski reagent (12 g of FeCl_3_ per L in 7.9 M H_2_SO_4_) (Glickmann and Dessaux, 1995). Bacterial isolates were inoculated in LB medium supplemented with the precursor DL-Tryptophan (0.01%) and as well in LB medium without DL-Tryptophan, incubated at 30°C for 72 h, with shaking at 180 rpm. After incubation, the cultures were centrifuged at 4°C for 10 min (6,000 rpm). Equals volume of supernatant and Salkowski reagent were mixed and incubated in the dark for 30 min, then assessed for color change. Development of reddish color indicated the presence of IAA. Optical density was taken at 540 nm by using UV/visible spectrophotometer. Standard curve of IAA was used to measure the concentration of IAA produced. The strains were quantitatively assessed for siderophores production using CAS (Chrome Azurol Sulphonate) reagent (Schwyn and Neilands, 1987). Supernatant of each bacterial culture was added in separate wells of microplate followed by the addition of 100 μL CAS reagent. After 20 min optical density was taken at 620 nm using microplate reader. Siderophore production by *K. variicola* UC4115 and *A. brasilense* Sp7 was measured in percent siderophore unit (psu) which was calculated according to the following formula: [(Ar-As)/Ar] ×100 = % siderophore units. Where, Ar = absorbance of reference (CAS solution and uninoculated broth), and As = absorbance of sample (CAS solution and cell-free supernatant of sample). The two strains were also screened for antifungal activities against *Sclerotinia sclerotiorum* (DSM 1946) using dual culture assay (Dikin et al., 2006), on potato dextrose agar (PDA) (Oxoid, Basingstoke, UK). The antagonistic activity was observed by measuring the size of the growth inhibition zone and the percentage of growth inhibition (PGI) was calculated using the formula: [(KR–R1)/KR] ×100 = % growth inhibition. Where, KR represents the colony diameter of the pathogen in the control plate, and R1 represents the colony diameter in the treated plate.

### Whole Genome Sequencing and Analysis

#### Genome DNA Extraction and Sequencing

Genomic DNA of *K. variicola* UC4115 was extracted from exponential phase LB broth culture using the E.Z.N.A Bacterial DNA kit according to the manufacturer's instruction (Omega Bio-tek, Georgia, USA). Genomic DNA was sequenced at Fasteris (Geneve, Switzerland) using an Illumina MiSeq operating with V3 chemistry in 300X2 bp paired-reads. Basecalling was performed with MiSeq Control Software 2.6.2.1, RTA 1.18.54.0, and bcl2fastq2 v2.17.1.14.

#### Genome Assembly and Annotation

Genome assembly was performed using PATRIC Unicycler v0.4.8 assembly pipeline (Wick et al., 2017). Genome annotation was performed both with RAST server (Aziz et al., 2008) and PATRIC RASTtk-enabled Genome Annotation Service (Brettin et al., 2015). The gene functions were further analyzed by BLASTP using Kyoto Encyclopedia of Genes Genomes (KEGG) database. The presence of plasmids was assessed by PlasmidFinder 1.3 (Carattoli et al., 2014). Phylogenetic tree was build using the codon trees pipeline in PATRIC which uses the amino acids and nucleotides sequences from PATRIC's global families (PGFams), that cross the genus boundary (Davis et al., 2016).

The genome sequence of *A. brasilense* Sp7 was downloaded from NCBI-Genomes database at https://www.ncbi.nlm.nih.gov/genome/. As for *K. variicola* UC4115, genome annotation was performed both with RAST server and PATRIC RASTtk-enabled Genome Annotation Service.

### Analysis of Hemolysin Phenotype

To evaluate the hemolytic properties, *K. variicola* UC4115 was aerobically cultured on Blood Agar plates (Sigma-Aldrich, Germany) containing 7% (w/v) defibrinated horse blood (EO Labs, Burnhouse, Scotland) at 30°C, overnight. *Staphylococcus aureus* ATCC 6538 was used as a positive control for hemolysis. The development of a clear or greenish zone around the colonies is indicative of the presence of β- or α-hemolysis (Patrone et al., 2020).

### Greenhouse Experiments

#### Inoculum's Preparation

Bacterial cultures of *K. variicola* UC4115 and *A. brasilense* Sp7 were inoculated in LB broth and incubated at 28–30°C with shaking at 180 rpm. After 14 h, cultures broths were centrifuged at 6,000 rpm for 10 min. The pellets were re-suspended in sterile distilled water (SDW) and washed thrice. The washed bacterial pellets were then reconstituted with SDW to obtain a turbid solution, whose optical density at 600 nm was adjusted to obtain a final density of 1 × 10^7^ CFU mL^−1^. These solutions were used for greenhouse inoculations.

#### Seedling Assays

The effects of bacterial inoculation were observed over two separated greenhouse experiments: the first one using tomato variety HEINZ 3402 in a conventional commercial peat and the second one using variety HEINZ 1301 in an organic commercial peat. Details about composition of media are reported in the Supplementary Table 1. For each experiment, 280 tomato seeds were sown in a polystyrene multicell flats (280 inverted pyramid cells, 13 cm^3^ volume), filled with the specific commercial soil, and covered with a layer of vermiculite. Seedlings were grown under typical greenhouse growing conditions (temperatures of 18°C at night and 24°C at day, 80% relative humidity). Seedlings were irrigated daily and no fertilizer was used. Both greenhouse experiments were designed as split plot, with six replicates. Main factor was bacterial inoculation (hereafter, inoculation), with three levels: (i) *K. variicola* UC4115, (ii) *A. brasilense* Sp7 (DSMZ 1690), plus (ii) a negative control with un-inoculated seedlings. Secondary factor was the type of application (hereafter, application), with three levels: application (i) at seed level, (ii) at root level, and at (iii) both seed and root (seed plus root) level.

For seed application, tomato seeds were wetted with 10 mL of bacterial inoculum, prepared as previously described, immediately after sowing, while for root application 10 mL of bacterial suspension was applied as soil drench on the rhizosphere of 3 week-old tomato seedlings. In the third application tested, bacterial inoculation of both seeds and roots were performed as described above. In the negative control thesis, the same volume (10 mL) of sterile distilled water was used. For application at seed level, replicates were taken off from six different cells with inoculated and controls seedlings at weeks 3, 4, and 5 after sowing, corresponding to the BBCH phenological stages 11–12, 12–13, and 13–14. For application at root and at seed plus root levels, samplings from six different cells with inoculated and controls seedling were performed at weeks 4, 5, and 6 after sowing, corresponding to BBCH 12–13, BBCH 13–14, BBCH > 14. After cutting off the aerial part of the seedlings, rhizosphere soil was removed with water added with Tween 80 (0.01% v/v), in order to facilitate the separation of roots from soil. Dry weights of separated roots and shoots were weighted after heating at 105°C, overnight.

### Root Characterizations

Soil samples were stored at 4°C until root separation and analysis were carried out. Determination of Root Length Density (RLD, cm cm^−3^) and root diameter were performed with the software winRHIZO *Reg* 2012. The Diameter Class Length (DCL, mm cm^−3^) was calculated for very fine (≤ 0.075 mm), fine (0.075–0.2 mm), medium (0.2-1.0 mm) and coarse (>1 mm) diameters, as adapted from Fiorini et al. (2018).

### Statistical Analyses

Data on the evolution over time of tomato seedlings above-ground and below-ground biomass in both experiments (i.e., conventional and organic) were subjected to analysis of variance (ANOVA) with a mixed-effect model using the “nlme” package of RStudio3.3.3 (Pinheiro et al., 2017). Phenological stages (BBCH) were included in the model as fixed factor while block effect was considered as random. Repeated measures were used to assess the effects of inoculation with *K. variicola* UC4115, *A. brasilense* Sp7 and negative control at seed level, root level, and seed plus root level over time. Since BBCH stages for application at seed level were different from those for application at root and at seed plus root level, analyses were performed separately for each type of application.

Data on the final effect of inoculation on root length density (RLD), and diameter class length (DCL) for very fine (Ø ≤ 0.075 mm), fine (Ø = 0.075–0.2 mm), medium (Ø = 0.2–1.0 mm), and coarse (Ø ≥ 1.0 mm) diameters were statistically analyzed with split-plot ANOVA.

When normality of variances was not confirmed using the Sharpiro-Wilk test, data were log transformed before analysis. Mean values were separated with Tukey honestly significant difference (HSD) test (α = 0.05), using the “Estimated Marginal Means, aka Least-Squares Means” package, version 1.2.4 (Lenth et al., 2018).

## Results

### Phenotypic Features of the Two Tested PGPR

For both strains *K. variicola* UC4115 and *A. brasilense* Sp7 the growth of a veil-like pellicle near the surface of the culture medium NFb was observed, moreover, the color change of the medium from green-blueish to blue was another indicator for the bacterial growth (Table 1). The capability of both strains to grow on nitrogen-free medium indicated their putative activity as diazotrophic bacteria.

**Table 1:** Plant growth promotion properties of *K. variicola* UC4115 and *A. brasilense* Sp7.

**PGP property**	***K. variicola* UC4115**	***A. brasilense* Sp7**
Growth on N-free agar medium	Growth and change color observed	Growth and change color observed
Phosphate solubilization	Level 2	–
IAA production (w/Try; w/o Try)	68.32 μg/ml; 3.16 μg/ml	3.06 μg/ml; 3.18 μg/ml
Siderophore production	41.50 psu	1.90 psu
Biocontrol activity against *Sclerotinia sclerotiorum*	33.33%.	41.54%

The isolate *K. variicola* UC4115 was able to solubilize the phosphate of the GY/Tricalcium phosphate medium by producing clear zone around the colonies after 7 days of incubation. Considering the halo's diameter bigger than 0 cm up to 1 cm, *K. variicola* strain UC4115 was classified as Level 2 phosphate solubilizer (Table 1). *A. brasilense* Sp7 strain was not able to grow on GY/Tricalcium phosphate medium (Table 1), so its capability to solubilize phosphate was not evaluated *in vitro*.

To screen for indole-3-acetic acid (IAA) production the Salkowski reagent was used, which gave different degree of red to the solution according to the different levels of IAA produced. The concentration of IAA produced by *K. variicola* strain UC4115 showed variation in presence of L-Tryptophan (68.32 μg mL^−1^) and without the IAA precursor tryptophan (3.16 μg mL^−1^) (Table 1). The concentration of IAA produced by strain *A. brasilense* Sp7 was very low both in presence (3.06 μg mL^−1^) and in absence (3.18 μg mL^−1^) of the IAA precursor tryptophan (Table 1).

The quantitative estimation of siderophore production was carried out using CAS reagent. Optical density revealed that the concentration of siderophore produced by *K. variicola* UC4115 measured 41.50 psu, while measured 1.90 psu for *A. brasilense* Sp7 (Table 1).

Antifungal activity, of both strains *K. variicola* UC4115 and *A. brasilense* Sp7, was tested against *Sclerotinia sclerotiorum* (DSM 1946) using the dual plate technique, showing a percentage of growth inhibition (PGI) corresponding to 33.33% for *K. variicola* UC4115, and 41.54% for *A. brasilense* Sp7 (Table 1).

As a safety measure, the activity of hemolysins for *K. variicola* UC4115 were tested. However, neither β-hemolytic nor α-hemolytic phenotypes were detected on blood agar plates. In contrast, the positive control *S. aureus* ATCC 6538 exhibited the expected β-hemolysin activity (data not shown).

### Genomic Features of the Two Tested PGPR

**Figure 1:**
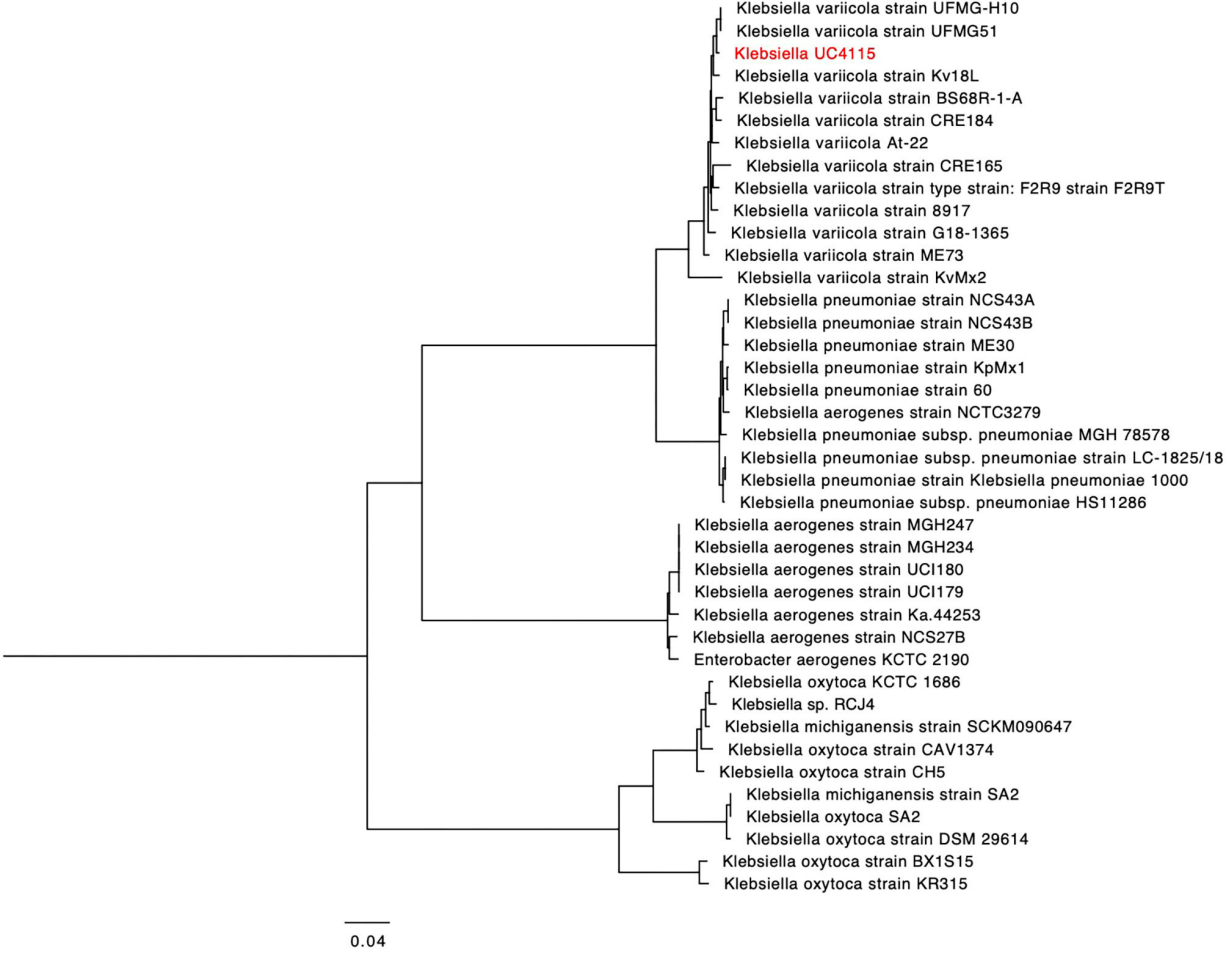
Phylogeny of *K. variicola* UC4115 based on the analysis of PGFams.

The general genomic properties of *K. variicola* UC4115 and *A. brasilense* Sp7 are presented in Table 2. The presence of one plasmid was verified using PlasmidFinder (v1.3), the plasmid was similar to plasmid pCAV1099-114 of *K. oxytoca* strain CAV1099, with 96% identity. We established a phylogenetic tree based on the PGFams, global proteins families that cross the genus boundary, with 100 conserved genes, zero deletions and zero duplications allowed. The tree supported the 16S results, confirming that the strain UC4115 is most closely related to *K. variicola* (Figure 1).

**Table 2:** General features of *K. variicola* UC4115 and *A. brasilense* Sp7 genomes.

**Feature**	***K. variicola* UC4115**	***A. brasiliense* Sp7**
Size (bp)	5,539,38 bp	7,100,241 bp
G + C content (%)	57.34	68.41
Number of CDSs	5,378	6,667
tRNA	77	74
rRNA	4	9
Plasmid	1	5
Coverage	309x	105x

Genome annotation was performed with PATRIC RASTtk-enabled Genome Annotation Service and further confirmed with RAST server. Relating to nitrogen fixation ability *K. variicola* UC4115 genome harbored *nifJHDKTYENXUSVWZMFLABQ* genes together with the *rnf* ABCDEG operon (Table 3). The genes *nifMFLJ*, and the *rnf* cluster, were absent from *A. brasilense* Sp7. However, *A. brasilense* Sp7 genome contained the *fixABCXJ* genes (Table 3).

**Table 3:** Genes annotation of *K. variicola* UC4115 and *A. brasilense* Sp7 genomes related to nitrogen fixation, phosphate solubilization, IAA production, siderophores synthesis, and biocontrol activity.

**Trait**	**PGfams ID**	**Gene annotation**	**KO/Gene_ID**	**KEGG_GENE_NAME**	**UC 4115**	**Sp7**
Nitrogenase complex	PGF_00025954	Nitrogenase iron protein	K02588	nifH	*	*
	PGF_00025951	Nitrogenase molybdenum-iron protein alpha chain	K02586	nifD	*	*
	PGF_00025953	Nitrogenase molybdenum-iron protein beta chain	K02591	nifK	*	*
	PGF_00120348	Putative nitrogen fixation protein FixT	K02593	nifT	*	*
	PGF_00025669	NifY protein	–	nifY	*	*
	PGF_00025964	Nitrogenase MoFe cofactor biosynthesis protein NifE	K02587	nifE	*	*
	PGF_00945843	Nitrogenase iron-molybdenum-cofactor biosynthesis protein NifN	K02592	nifN	*	*
	PGF_00025961/ PGF_00025962	Nitrogen fixation protein NifX	K02596	nifX	*	*
	PGF_00420050	Cysteine desulfurase NifS	K04487	nifS	*	*
	PGF_00072326	Homocitrate synthase	K02594	nifV	*	*
	PGF_01958698	Putative NifW protein	K02595	nifW	*	*
	PGF_00025671	Putative NifZ protein	K02597	nifZ	*	*
	PGF_01971790	Nitrogen fixation protein NifM	K03769	nifM	*	
	PGF_00071382	Flavodoxin FldA	K03839	nifF	*	
	PGF_00029105	Nitrogen fixation negative regulator NifL/PAS-PAC protein	K23916	nifL	*	
	PGF_03973235	Nif-specific regulatory protein	K02584	nifA	*	*
	PGF_06674514	Nitrogenase cofactor biosynthesis protein NifB	K02585	nifB	*	*
	PGF_00025976	Nitrogen fixation protein NifQ	K15790	nifQ	*	*
	PGF_00046007	Putative pyruvate:ferredoxin (flavodoxin) oxidoreductase	K03737	nifJ	*	
	PGF_00015530	Iron-sulfur cluster assembly scaffold protein	K04488	nifU	*	*
	PGF_00401722	Nitrogenase-associated protein NifO	–	nifO	*	*
Nitrogenase transport	PGF_00424131	Na(+)-translocating NADH-quinone reductase subunit E	K03617	rnfA	*	
	PGF_10542033	Electron transport complex protein RnfB; Required for nitrogen fixation	K03616	rnfB	*	
	PGF_04400591	Electron transport complex protein rnfC	K03615	rnfC	*	
	PGF_01678333	Electron transport complex protein RnfD; Required for nitrogen fixation	K03614	rnfD	*	
	PGF_01484108	NADH-ubiquinone oxidoreductase	K03613	rnfE	*	
	PGF_00424141	Electron transport complex protein RnfG	K03612	rnfG	*	
Electron transport	PGF_05015473	Ferredoxin-like protein FixX/4Fe-4S ferredoxin	K03855	FixX		*
	PGF_03136394	Electron transfer flavoprotein-quinone oxidoreductase FixC	K00313	FixC		*
	PGF_03134445	Electron transfer flavoprotein, alpha subunit FixB	K03522	FixB		*
	PGF_03098759	Electron transfer flavoprotein, beta subunit FixA	K03521	FixA		*
	PGF_00849787	Two-component system response regulator	K14987	FixJ		*
Gluconic acid	PGF_04577966	Quinoprotein glucose dehydrogenase/glucose dehydrogenase, PQQ-dependent	K00117	gcd	*	
	PGF_01393943	Pyrroloquinoline-quinone synthase C	K06137	pqqC	*	*
	PGF_01084777	Coenzyme PQQ synthesis protein F	–	pqqF	*	
	PGF_00418484	Coenzyme PQQ synthesis protein B	K06136	pqqB	*	*
	PGF_03579782	Coenzyme PQQ synthesis protein D	K06138	pqqD	*	
	PGF_00418486	Coenzyme PQQ synthesis protein E	K06139	pqqE	*	*
Phosphonate transporter	PGF_12684827	Phosphonate ABC transporter, permease protein	K02042	phnE1	*	
	PGF_00033841	Phosphonate ABC transporter, permease protein	K02042	phnE2	*	
	PGF_00033852	Phosphate-binding protein of phosphonate ABC transporter	K02044	phnD	*	
	PGF_00033832	Phosphonate transport system ATP-binding protein	K02041	PhnC	*	
Phosphate transporter	PGF_07668761	Phosphate transport system substrate-binding protein	K02040	Pst S	*	*
	PGF_01072302	Phosphate transport system permease protein	K02038	Pst A	*	*
	PGF_02405545	Phosphate transport system permease protein	K02037	Pst C	*	*
	PGF_06213055	Phosphate transport system ATP-binding protein	K02036	Pst B	*	*
Indole-3-acetic acid (IAA) biosynthesis	PGF_05599542	Indole-3-pyruvate decarboxylase	K04103	ipdC	*	*
	PGF_00049805	Amidase	K01426	–	*	
	PGF_07597988	Aldehyde dehydrogenase (NAD+) (EC 1.2.1.3)		aldh	*	*
	PGF_00418275	Nitrile hydratase, alpha subunit	K01721	nthA	*	*
	PGF_00418276	Nitrile hydratase, beta subunit	K20807	nthB	*	*
	PGF_03811905/ PGF_02254418	Histidinol-phosphate aminotransferase	K00817	hisC	*	*
Siderophore production	PGF_00015658/ PGF_00015659	Isochorismate/ Apo-aryl carrier protein	K01252	entB, dhbB, vibB, mxcF	*	*
	PGF_00025850/ PGF_07637567	2,3-dihydroxybenzoate-AMP ligase	K02363	entE, dhbE, vibE, mxcE	*	*
	PGF_05075091	Enterobactin synthetase component F	K02364	entF	*	*
	PGF_00424602	Enterobactin exporter	K08225	entS	*	
	PGF_00023831/ PGF_08225224	2,3-dihydro-2,3-dihydroxybenzoate dehydrogenase	K00216	entA	*	*
	PGF_00015696	Isochorismate synthase	K02361	entC	*	
	PGF_00422373	4′-phosphopantetheinyl transferase EntD	K02362	entD	*	
	PGF_00037591	Proofreading thioesterase in enterobactin biosynthesis	K24147	entH	*	
	PGF_07721642	MbtH-like protein	K05375	MbtH		*
	PGF_00004447	Ferric enterobactin-binding periplasmic protein	K23185	FepB	*	
	PGF_00004441	Ferric enterobactin transport system permease protein	K23186	FepD	*	
	PGF_00004444	Ferric enterobactin transport system permease protein	K23187	FepG	*	
	PGF_00004439	Ferric enterobactin transport ATP-binding protein	K23188	FepC	*	
	PGF_00424600/ PGF_08225224	Enterobactin esterase	K07214	Fes	*	*
	PGF_00057226	TonB-dependent receptor; Outer membrane receptor for ferric enterobactin and colicins B, D	K19611	FepA	*	*
	PGF_00052044	Alternative sigma factor	–	PvdS		*
	PGF_00045754	PvdE, pyoverdine ABC export system, fused ATPase and permease components	K06160	PvdE		*
4-hydroxybenzoate Production	PGF_00417843	Chorismate-pyruvate lyase	K03181	ubiC	*	
GABA	PGF_07204877	Succinate-semialdehyde dehydrogenase	K00135	gabD	*	*
	PGF_04337880	4-aminobutyrate aminotransferase	K07250	gabT	*	*
Phenazine biosynthesis	PGF_10329977	Phenazine biosynthesis	-	phzF	*	*

The symbol * indicates that the gene is present in the genome.

Regarding phosphorus solubilization ability, *K. variicola* UC4115 genome harbored *gcd* and *pqqBCDEF* genes. Moreover, phosphate (*PstABCS*) and phosphonate (*PhnCDE1E2*) transporter system genes were detected (Table 3). The genes *gcd, pqqFD*, and *phn* cluster were absent from *A. brasilense* Sp7 (Table 3).

Gene prediction identified genes specifically associated with IAA production. Indeed, both strains *K. variicola* UC4115 and *A. brasilense* Sp7 carried *ipdC, aldh*, and *nthAB* genes, the gene amidase was absent from *A. brasilense* Sp7 (Table 3).

Relating to the synthesis of siderophores *K. variicola* UC4115 carried *entABCDEFGHS* genes together with several genes for siderophores receptors such as *FepABCDG*. While, *A. brasilense* Sp7 harbored *PvdSE* genes (Table 3).

Regarding to biocontrol activities *UbiC, gabDT*, and *phzF* genes were detected in *K. variicola* UC4115 genome. All the defense genes detected in *K. variicola* UC4151 strain was also detected in *A. brasilense* Sp7 strain, with the exception of *UbiC* (Table 3).

### Seedling Assays

#### Evolution Over Time of Tomato Seedling Biomass Under Conventional Management

**Figure 2:**
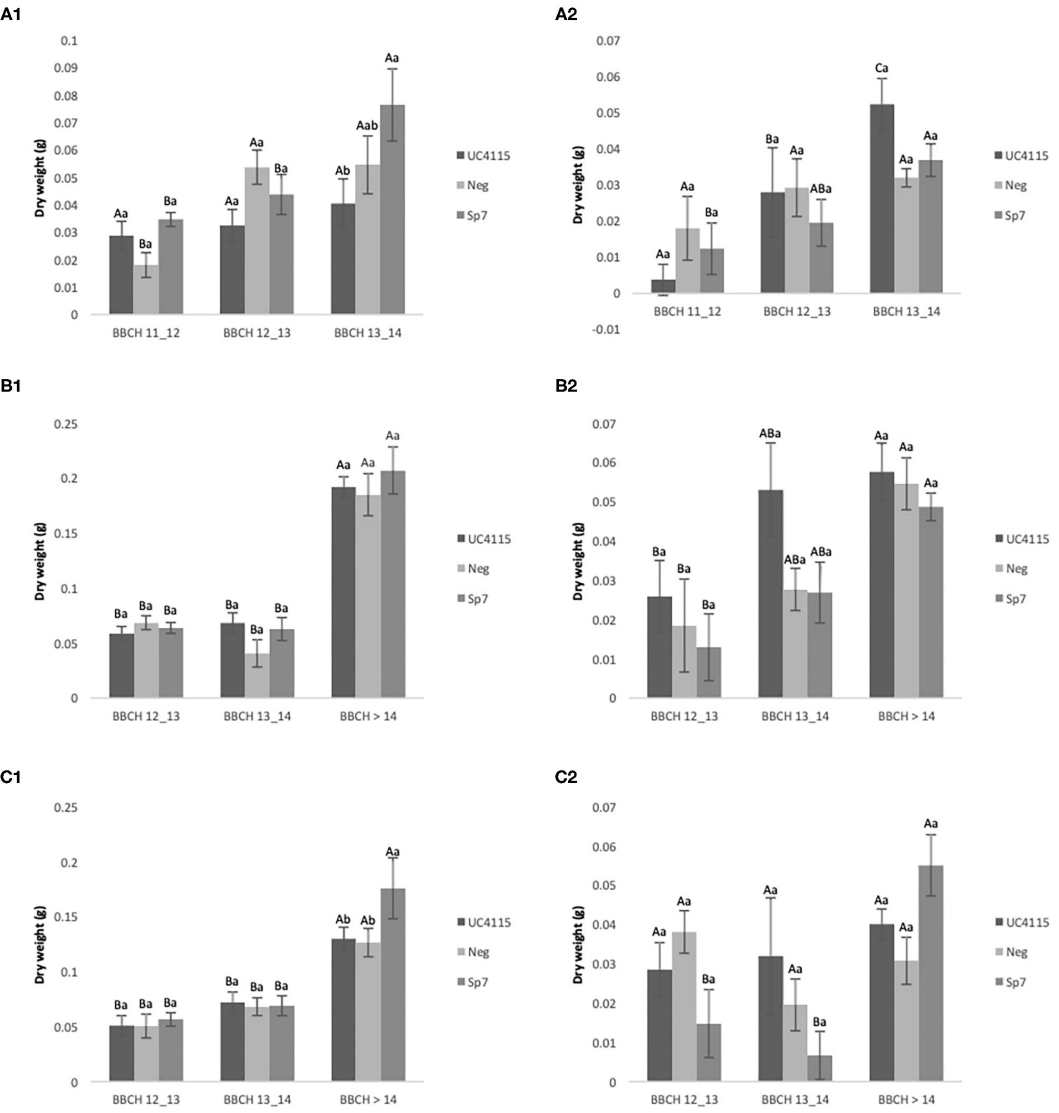
Conventional experiment. Evolution over time of tomato plant above-ground biomass **(A1,B1,C1)** and below-ground biomass **(A2,B2,C2)** after inoculation with UC4115, Sp7, and negative control at seed level **(A1,A2)**, root level **(B1,B2)**, and seed plus root level **(C1,C2)**. Mean values ± standard deviation. Capital letters indicated differences among physiological state (BBCH) of tomato plant within the same inoculation; lowercase letters indicate differences among different inoculations within the same physiological state.

After application at seed level, negative control and *A. brasilense* Sp7 increased above-ground biomass of tomato seedlings passing from BBCH 11–12 to BBCH 13–14. Conversely, *K. variicola* UC4115 did not lead to any above-ground biomass variation between the two phenological stages. The inoculation hierarchy at BBCH 13–14 was *A. brasilense* Sp7 ≥ negative control ≥ *K. variicola* UC4115 (Figure 2A1). Below-ground biomass of tomato seedlings was increased from BBCH 11–12 to BBCH 13–14 by *K. variicola* UC4115, while not by *A. brasilense* Sp7 and negative control. This turns into higher biomass in *K. variicola* UC4115 than in *A. brasilense* Sp7, while the negative control did not differ from both the former and the latter (Figure 2A2).

After application at root level, the above-ground biomass of tomato seedling was affected by all inoculations: *K. variicola* UC4115, *A. brasilense* Sp7 and negative control increased above-ground biomass from BBCH 12-13 to BBCH >14, and no difference between inoculations occurred at each phenological stage (Figure 2B1). A similar pattern was observed for the below-ground biomass of tomato seedlings, which increased with increasing BBCH stage (Figure 2B2). Also, after the application at seed and root level, *K. variicola* UC4115, *A. brasilense* Sp7 and negative control increased above-ground biomass of tomato seedling passing from BBCH 12–13 to BBCH > 14. Differences between inoculations occurred only at to BBCH >14, when *A. brasilense* Sp7 had higher above-ground biomass than *K. variicola* UC4115 and negative control (Figure 2C1). As regards below-ground biomass of tomato seedlings, a few differences were found between and within each BBCH stage: only *A. brasilense* Sp7 increased values from BBCH 12–13 to BBCH > 14 (Figure 2C2).

On overall, the results indicated that the inoculation at seed- level and the co-inoculation at seed plus root level were the more effective applications, while the root application had shown less evidence in terms of dry root and dry shoot biomass. Further, these data indicated that the effects, on root and shoot dry mass, are more evident when the inoculations were made at the latest stages of growth after sowing.

#### Evolution Over Time of Tomato Seedling Biomass Under Organic Management

**Figure 3:**
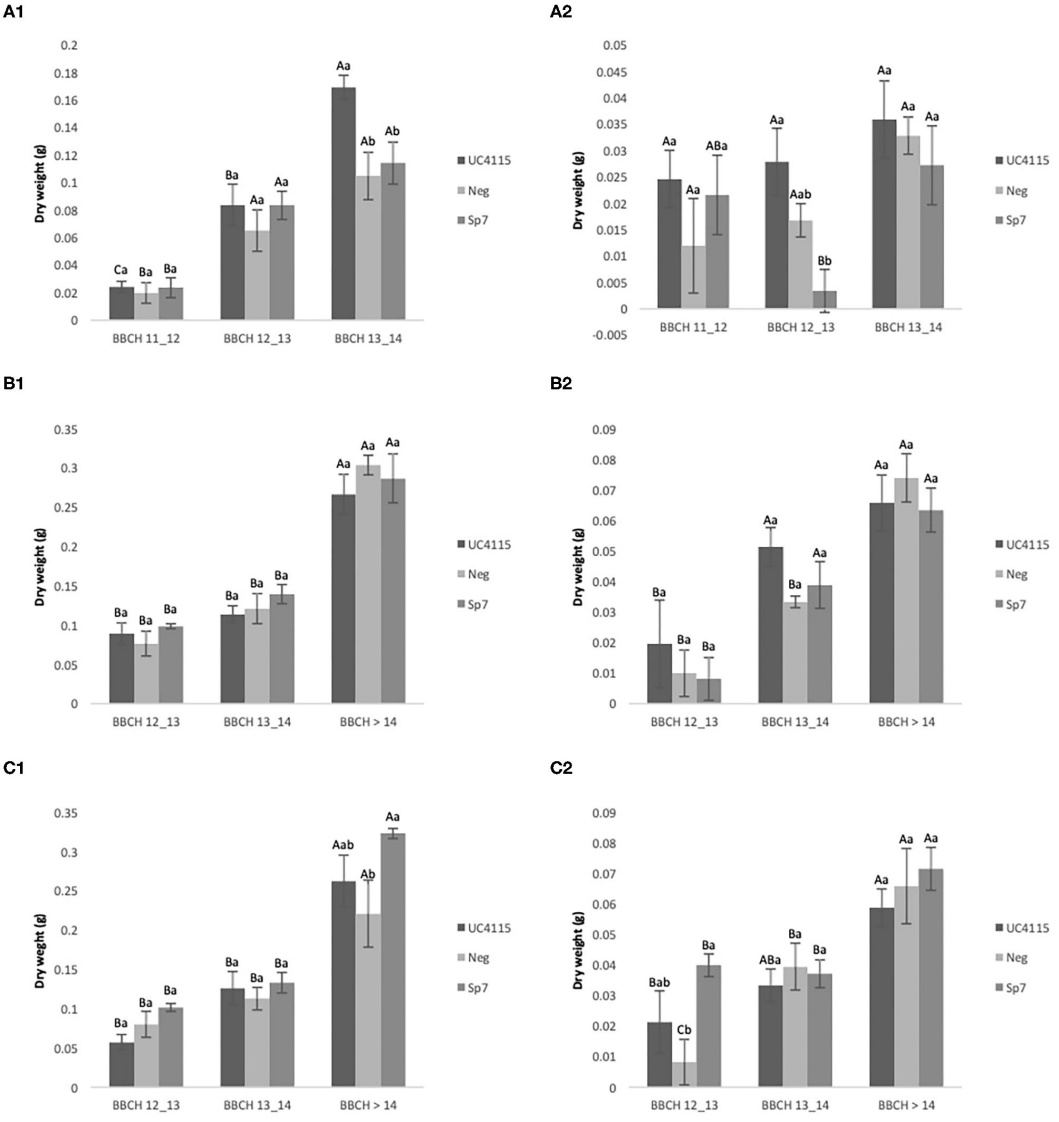
Organic experiment. Evolution over time of tomato plant above-ground biomass **(A1,B1,B2)** and below-ground biomass **(A2,B2,C2)** after inoculation with UC4115, Sp7, and negative control at seed level **(A1,A2)**, root level **(B1,B2)** and seed plus root level **(C1,C2)**. Mean values ± standard deviation. Capital letters indicated differences among physiological state (BBCH) of tomato plant within the same inoculation; lowercase letters indicate differences among different inoculations within the same physiological state.

After application at seed level, all inoculations increased above-ground biomass of tomato seedling passing from BBCH 11–12 to BBCH 13–14. Differences between inoculations occurred at BBCH 13–14, when *K. variicola* UC4115 had higher biomass than *A. brasilense* Sp7 and negative control, where the biomass increment was 62% when compared to negative control (Figure 3A1). Trends of below-ground biomass of tomato seedlings did not show a specific pattern and differences between inoculations were found only at BBCH 13–14, when *A. brasilense* Sp7 was lower than *K. variicola* UC4115 and negative control (Figure 3A2).

After application at root level, the above-ground and below-ground biomasses of tomato seedling were not significantly affected by inoculations at any BBCH stage (Figures 3B1,B2). *K. variicola* UC4115, *A. brasilense* Sp7 and negative control increased both biomass fractions from BBCH 12–13 to BBCH > 14. Similarly, above-ground biomass of tomato seedling was increased from BBCH 12–13 to BBCH > 14 by all inoculations, after application at seed and root level. However, *A. brasilense* Sp7 induced higher biomass than Neg at BBCH > 14, while *K. variicola* UC4115 did not differ from both the former and the latter (Figure 3C1). Also, below-ground biomass increased passing from BBCH 12–13 to BBCH > 14 in all inoculations. Nevertheless, differences here were found at BBCH 12–13, when negative control was lower that *A. brasilense* Sp7 (Figure 3C2).

Even under organic management the inoculation at seed level and the co-inoculation at seed plus root level, were more effective than the root application, that had shown no evidence in terms of dry root and shoot mass.

#### Root Length Density (RLD) and Diameter Class Length (DCL) of Tomato Seedlings at the End of Organic Management

Root length density of tomato seedlings at the end of the experiment was significantly affected by inoculation, while not by application. In detail, *K. variicola* UC4115 had on average higher RLD than *A. brasilense* Sp7, while negative control did not differ from the former and the latter. The interaction I × T was also significant and *K. variicola* UC4115 with application at root level had higher RLD than *A. brasilense* Sp7 with application at seed- and root level. All the others were in the between (Table 4).

**Table 4:** Root length density (RLD), and diameter class length (DCL) for very fine (Ø = 0.00–0.075 mm), fine (Ø = 0.075–0.2 mm), medium (Ø = 0.2–1.0 mm), and coarse (Ø = > 1.0 mm) diameters for organic experiment, as affected by inoculation (I) and application (A).

			**DCL (cm cm**^****–3****^**)**				
**Source of variation**	**Inoculation**		**RLD (cm cm^**–3**^)**	**Ø = 0.00–0.075 mm**	**Ø = 0.075–0.2 mm**	**Ø = 0.2–1.0 mm**	**Ø = > 1.0 mm**
Inoculation	UC4115		300.105^a^	23.047	81.870	192.533^a^	2.655^ab^
	Neg		284.640^ab^	21.475	81.851	177.983^ab^	3.331^a^
	Sp7		261.074^b^	20.759	75.530	162.712^b^	2.074^b^
*P-value*			<0.01	–	–	<0.01	<0.05
			**DCL (cm cm**^**–3**^**)**				
**Source of variation**	**Application**		**RLD (cm** **cm**^**–3**^**)**	**Ø** **=** **0.00–0.075 mm**	**Ø** **=** **0.075–0.2 mm**	**Ø** **=** **0.2–1.0 mm**	**Ø** **=** **>** **1.0 mm**
Application	S		285.964	22.824	78.106	182.047	2.987
	R		292.869	21.923	86.415	181.684	2.847
	SR		266.986	20.535	74.730	169.495	2.226
*P-value*			–	–	–	–	–
			**DCL (cm cm**^**–3**^**)**				
**Source of variation**	**Inoculation**	**Application**	**RLD (cm cm**^**–3**^**)**	**Ø** **=** **0.00–0.075 mm**	**Ø** **=** **0.075–0.2 mm**	**Ø** **=** **0.2–1.0 mm**	**Ø** **=** **>** **1.0 mm**
I × A	UC4115	S	308.273^ab^	22.464	78.140	204.094^a^	3.574
		R	322.774^a^	24.495	91.952	203.985^a^	2.342
		SR	269.268^ab^	22.182	75.516	169.520^ab^	2.050
	Neg	S	280.265^ab^	22.976	83.701	170.836^ab^	2.752
		R	288.240^ab^	21.612	88.628	173.933^ab^	4.068
		SR	285.414^ab^	19.839	73.224	189.179^ab^	3.172
	Sp7	S	269.354^ab^	23.033	72.476	171.212^ab^	2.634
		R	267.592^ab^	19.661	78.664	167.136^ab^	2.131
		SR	246.276^b^	19.583	75.450	149.787^b^	1.456
*P-value*			<0.05	–	–	<0.05	–

Values followed by the same letter in the each column are not statistically significant according to Tukey's HSD test.

Diameter class length for very fine and fine roots was never affected by inoculation or application, as well as by the interaction I × T. Significant differences were found in DCL for medium (with Inoculation and I × T as sources of variation) and coarse roots (with Inoculation as source of variation): (i) Inoculation as source of variation showed higher DCL for medium roots with *K. variicola* UC4115 than with *A. brasilense* Sp7, while I × T showed higher DCL for medium roots under *K. variicola* UC4115 with application at seed-, and at root level than under *A. brasilense* Sp7 with seed plus root level; (ii) coarse roots had higher DCL on average under negative control than under *A. brasilense* Sp7, while *K. variicola* was in the between (Table 4).

#### Root Length Density (RLD) and Diameter Class Length (DCL) of Tomato Seedlings at the End of Conventional Management

Inoculation significantly affected RLD of tomato seedlings at the end of the experiment. In detail, *K. variicola* UC4115 and *A. brasilense* Sp7 had on average higher RLD than negative control. Also, the interaction I × T was found to be significant: RLD under *K. variicola* UC4115 with application at root-, and at seed plus root level, and under *A. brasilense* Sp7 with application at root level was higher than that under negative control with application at the root level. All the other combinations were in the between (Table 5).

**Table 5:** Root length density (RLD), and diameter class length (DCL) for very fine (Ø = 0.00–0.075 mm), fine (Ø = 0.075–0.2 mm), medium (Ø = 0.2–1.0 mm), and coarse (Ø = > 1.0 mm) diameters for conventional experiment, as affected by inoculation (I) and application (A).

			**DCL (cm cm**^****–3****^**)**				
**Source of variation**	**Inoculation**		**RLD (cm cm^**–3**^)**	**Ø = 0.00–0.075 mm**	**Ø = 0.075–0.2 mm**	**Ø = 0.2–1.0 mm**	**Ø = > 1.0 mm**
Inoculation	UC4115		140.844^a^	26.150	32.763	81.217	0.714
	Neg		107.470^b^	19.881	27.110	60.063	0.417
	Sp7		134.758^a^	22.980	32.320	78.700	0.759
*P-value*			<0.01	–	–	–	–
			**DCL (cm cm**^**–3**^**)**				
**Source of variation**	**Application**		**RLD (cm** **cm**^**–3**^**)**	**Ø** **=** **0.00–0.075 mm**	**Ø** **=** **0.075–0.2 mm**	**Ø** **=** **0.2–1.0 mm**	**Ø** **=** **>** **1.0 mm**
Application	S		120.514	18.973	29.787	696.694	0.794
	R		128.489	24.865	31.078	732.041	0.633
	SR		134.070	25.173	31.328	771.058	0.463
*P-value*			–	–	–	–	–
			**DCL (cm cm**^**–3**^**)**				
**Source of variation**	**Inoculation**	**Application**	**RLD (cm cm**^**–3**^**)**	**Ø** **=** **0.00–0.075 mm**	**Ø** **=** **0.075–0.2 mm**	**Ø** **=** **0.2–1.0 mm**	**Ø** **=** **>** **1.0 mm**
I × A	UC4115	S	114.228^ab^	14.089	28.808^ab^	71.331^ab^	0.000^b^
		R	151.681^a^	34.438	34.509^a^	81.894^a^	0.840^ab^
		SR	156.624^a^	29.924	34.973^a^	90.425^a^	1.301^a^
	Neg	S	125.262^ab^	18.933	32.325^ab^	72.831^ab^	1.174^a^
		R	77.398^b^	20.219	18.699^b^	38.442^b^	0.037^b^
		SR	119.751^ab^	20.491	30.305^ab^	68.915^ab^	0.040^b^
	Sp7	S	122.052^ab^	23.898	32.100^ab^	64.846^ab^	1.208^a^
		R	156.387^a^	19.936	36.153^a^	99.276^a^	1.022^a^
		SR	125.836^ab^	25.105	28.707^ab^	71.977^ab^	0.046^ab^
*P-value*			<0.01	–	<0.05	<0.01	<0.001

Values followed by the same letter in the each column are not statistically significant according to Tukey's HSD test.

The interaction I × T significantly affected also the response of DCL for fine, medium, and coarse roots: in any case *K. variicola* UC4115 with application at seed- and root level had the highest DCL, while negative control with application at the root level the lowest (Table 5).

## Discussion

### Phenotypic and Genomic Features of the Two Tested PGPR

In the current study *K. variicola* UC4115 was isolated from the rhizosphere and rhizoplane soil of tomato plants, from a field with a long history of reduced tillage plus cover crops management. The isolate was screened for biocontrol and PGP activities according to Guerrieri et al. (2020), showing high growth-promoting and defense abilities. We performed a functional annotation of genes contributing to the five plant-beneficial functions previously tested *in-vitro*. Furthermore, the *in-vitro* characterization, the whole genome sequencing and annotation, performed with *K. variicola* UC4115, were compared with the well-known PGPR *Azospirillum brasilense* strain Sp7 (GenBank: GCA_001315015.1). *Azospirillum* sp. is able to colonize hundreds of plant species and significantly improves their growth, development and productivity (Cassán and Diaz-Zorita, 2016). In detail strain *A. brasilense* Sp7 is well-characterized free-nitrogen fixing bacteria, particularly known for its anchoring capabilities at root surface (Ramirez-Mata et al., 2018). Hence, this global comparative study allowed to increment the knowledge about the newly isolated strain *K. variicola* UC4115 and to observe how different genera of PGPR affect differently the plant host.

According to the functional annotation, *K. variicola* UC4115 strain contained a number of genes corroborating the *in-vitro* results. Both strains *K. variicola* UC4115 and *A. brasilense* Sp7 were able to grown on nitrogen-free medium, this indicates their putative activity as diazotrophic bacteria. Indeed, according to literature both species are among the major nitrogen-fixing bacteria detected in root system (Bhattacharjee et al., 2008). Nitrogenase is the enzyme that catalyzes the conversion of atmospheric N_2_ to a bio-accessible form of nitrogen, and it consists of Fe-protein encoded by *nifH* (component I) and MoFe-protein encoded by *nifDK* (component II). Furthermore, the gene cluster from *Klebsiella* genome has been a model system for studying nitrogen fixation and consists in a total of 20 genes, *nifJHDKTYENXUSVWZMFLABQ* (Rubio and Ludden, 2008). The *K. variicola* UC4115 genome contains all the above *nif* genes together with the *rnf* ABCDEG operon, which encodes a putative membrane-bound complex related to electron transport to nitrogenase (Jeong and Jouanneau, 2000). Even if, genes *nifMFLJ*, and the *rnf* cluster, were absent from *A. brasilense* Sp7, this strain genome contained the *fix* genes (*fixABCXJ*) that are essential for nitrogen fixation but do not have an homologous counterpart in *Klebsiella* genus (Fischer, 1994). *Fix* genes were especially detected in rhizobia such as *Bradyrhizobium japonicum* and *Azorhizobium caulinodans* (Tsoy et al., 2016). In detail, it has been discovered that the *fixABCX* gene products probably replace the missing *NifJ* and *NifF* electron transfer proteins in rhizobia, that operate in *Klebsiella*. Moreover, it was previously suggested that *nif* gene number might vary according to the physiology of a bacterium. Indeed, alternatively, unidentified proteins might replace the missing *Nif* products (Masson-Boivin et al., 2009).

The capability of *K. variicola* UC4115 to solubilize the insoluble source of phosphate, contained in the GY/Tricalcium medium, was further confirmed through the identification of genes associated with phosphate solubilization. Gluconic acid (GA) is an organic acid recognized as one of the major responsible for the solubilization of mineral phosphate in most bacteria. GA biosynthesis is catalyzed by glucose-1-dehydrogenase (GDH) and its co-factor pyrrolo-quinolone quinine (PQQ) (Ramachandran et al., 2006). The *K. variicola* UC4115 genome possesses several genes related to GA biosynthesis and its co-factor genes, including *pqqBCDEF* (Wagh et al., 2014), while the gene *pqqA* is lacking. Researchers (Liu et al., 2016), during the study of the genome of the PGPR *Klebsiella sp*. D5A, have already reported the lacking of this specific gene. The enzyme encoded by *pqqA* would appear not essential for the biosynthesis of PQQ (Toyama and Lidstrom, 1998). Furthermore, the uptake of the inorganic P in *K. variicola* UC4115 may be promoted by the high affinity with the phosphate transporter system, *PstABCS*, and the phosphonate transporter system, *PhnCDE1E2* (Liu et al., 2016; Shariati et al., 2017), indeed various studies have indicate that phosphonates are another rich source of soil P (Oliverio et al., 2020). The genes *gcd, pqqFD* and *phn* cluster were absent from *A. brasilense* Sp7. Moreover, *A. brasilense* Sp7 strain was not able to grow on GY/Tricalcium phosphate medium, so its capability to solubilize phosphate was not evaluated *in vitro*. Usually the *pqqBCDE* are very conservative genes shared by different type of PGPR, indeed, PQQ is a co-factor implicated in several cellular process (Bruto et al., 2014). However, the incapability of *Azospirillum sp*. to solubilize P since they lack the *pqq* genes was already evaluated (Vikram et al., 2007).

According to literature the IAA-producing potential of species *K. variicola* is another dominant feature as PGPR, together with nitrogen fixation (Kim et al., 2017). In Trp-dependent IAA biosynthesis, four pathways have been postulated: (i) the indole-3-acetamide (IAM) pathways; (ii) the indole-3-pyruvate (IPA) pathways; (iii) the tryptamine (TAM) pathways; and (iv) the indole-3-acetonitrile (IAN) pathway (Spaepen and Vanderleyden, 2011). Here, two proposed pathways, the IAN and IPA pathways, are identified in the genome of *K. variicola* UC4115. Indeed, the isolate carries the nitrile hydratase (*nthAB*) and the amidase genes, which contribute, respectively, to the conversion of indole-3-acetonitrile (IAN) in indole-3-acetamide (IAM) and finally in IAA, and the *ipdC* gene, the key enzyme for the indole-3-pyruvate (IPA) decarboxylation (Spaepen and Vanderleyden, 2011). These two pathways have been also detected in the genome of the PGPR *Klebsiella sp*. D5A (Liu et al., 2016). The presence of *ipdC* gene in the *A. brasilense* Sp7 genome confirm the presence of the IPA pathways. Nonetheless, the concentration of IAA produced by strain *A. brasilense* Sp7 was very low both in presence (3.06 μg mL^−1^) and in absence (3.18 μg mL^−1^) of the IAA precursor tryptophan. However, in 1992 Bar and Okon, found out that indole-3-acetic acid production of *A. brasilense* Sp7 depended on culture age and amount of tryptophan supplement (Bar and Okon, 1992). During our study we only tested one concentration of tryptophan at 0.01%, to analyze the effective capability of producing IAA, several concentrations should be compared.

Relating to the siderophores production, *K. variicola* UC4115 carried genes *entABCDEFGH* which catalyze the conversion of chorismate, an intermediate of aromatic amino acid synthesis, into the enterobactin (Hubrich et al., 2021), and the gene *entS*, which is responsible for the transport of the siderophore. Furthermore, *K. variicola* strain UC4115 encodes several genes for siderophores receptors (*FepBCDG*), including *TonB*-dependent receptor outer-membrane receptor (*FepA*) (Shariati et al., 2017). The genes *entS, entCDH*, and the *Fep* cluster were absent from *A. brasilense* Sp7. This seemed to confirm the extremely low value (1.90 psu), of siderophore produced by the strain *A. brasilense* Sp7. However, according to literature *A. brasilense* strain Sp7 seems to be able to secrete a catecholtype siderophore called spirilobactin, that can chelate and transport iron (Alahari et al., 2006; Tortora et al., 2011). Moreover, our genomic study did not detect the presence of genes involved in pyoverdine synthesis (*Pvd*), one of the major class of siderophores (Liu et al., 2016), in *K. variicola* UC4115 genome, by contrast *PvdSE* were detected in *A. brasilense* Sp7 genome.

Biocontrol activities are important mechanisms by which plant growth promoting bacteria suppress plant pathogens (Shen et al., 2013). It has been stated that PGPR may produce molecules able to suppress plant pathogenic microbes or are responsible for pest and disease inhibition. The common compound produced by PGPR and major studied so far are 4-hydroxybenzoate and γ-aminobutyric acid (GABA). *UbiC* is the involved in 4-hydroxybenzoate synthesis, while *gabD* and *gabT* genes contribute to GABA synthesis (Gupta et al., 2014). Despite of the low biocontrol activity against *S. sclerotiorum, K. variicola* UC4115 genome contains all the above genes. Moreover, also the gene *phzF*, involved in the synthesis of the antibiotic phenazine (Gupta et al., 2014) was detected. The higher biocontrol activity of *A. brasilense*, against *S. sclerotiorum*, could be explain by the production of the siderophore pyoverdine, indeed the biocontrol activity of these molecules seems to be involved in the biocontrol activity of *Pseudomonas fluorescens* (Shen et al., 2013).

### Seedling Assays

Under organic management, *K. variicola* UC4115 showed the greatest performance at seed level. The effects were always evident at the latest stages of growth after sowing. The increment of dry shoot biomass extended both phenotypic and genomic characterization, underlying its capability as nitrogen-fixing rhizobacteria, phosphate solubilizers, and siderophores producer, that are those abilities that confer a major impact on the above-ground biomass. Moreover, this result could also be affected by the greatest influence, of *K. variicola* UC4115, on length and diameters of the roots. This is certainty due to the significant amount of IAA produced by the strain confirmed by the genome analysis and *in-vitro* screening. Auxin can regulate the size of the root apical meristems (Aloni et al., 2006). In detail, IAA helps in the production of longer roots with increased number of root hairs and root laterals which are involved in nutrient uptake (Mohite, 2013). The IAA effects seemed more evident after application at root- and seed-level. Data at root-level seemed to show a negative tendency between biomass values and RLD and DCL values. Presumably the greater RLD was due to the increased root thickness, and RLD did not translate into high root and shoot biomass (Nada and Abogadallah, 2018).

The capability of *A. brasilense* Sp7 to increment the dry shoot biomass under conventional management, at both seed and seed-root application, reflected its high value trait as nitrogen-fixing rhizobacteria and underlined the genomic characterization. Indeed, several studies stated the importance of the role of *fixABC* cluster and some of the *nif* genes in the *A. brasilense* Sp7 as high nitrogen-fixing rhizobacteria (Galimand et al., 1989). Furthermore, Jankiewicz, in 2006, observed how magnesium ions present in the bacterial growth medium of *Pseudomonas*, improved the synthesis of siderophore pyoverdine (Jankiewicz, 2006), by assumption, the Mg within the conventional peat, could have stimulated the *Pvd* genes detected in *A. brasilense* Sp7 genome. Moreover, the higher performance of *A. brasilense* Sp7 compared to *K. variicola* UC4115, could be explained by the presence of fertilizers in the commercial peat, hence, the positive effects of association between strains of *A. brasilense* inoculations and N rates, are frequently reported in literature (Marini et al., 2015). In addition, undoubtedly, interactions between plant genotype and bacteria, are one of the most contributing factors to the complexity of responses to inoculation (Rodriguez et al., 2019). The capability of *A. brasilense* Sp7 to affect the RLD and DCL after root application, confirmed the genomic evaluation performed and the presence of IPA pathway, despite of the *in-vitro* results. This was in accord with the scientific literature about the IAA-producing potential of strain *A. brasilense* Sp7 (Castro-Guerrero et al., 2012). For both *K. variicola* UC4115 and *A. brasilense* Sp7 we observed a negative tendency between biomass and RLD and DCL parameters.

## Conclusion

In conclusion, in this study, we present a comprehensive genomic and greenhouse evaluation of PGPRs, using the multifunctional *K. variicola* UC4115 as a case study. The screening *in-vitro* results were confirmed and supported by the genome analysis, demonstrating that *K. variicola* UC4115 acted as PGPR, indeed the strain contained many of the signature genes that are functionally linked to the plant growth promotion traits. Comparative *in-vitro* screening and genomic analyses with the well-known PGPR *A. brasilense* Sp7 revealed different accumulation of PBFC genes, and potential PGPR features not detected through the *in-vitro* assay. Comparative greenhouse assays on tomato seedling allowed to observed how these differences affect the plant growth. Here, we report for the first time a holistic comparative discussion between phenotypic analysis, genomic characterization and plant study. The comparison between functional annotation and *in-vivo* results, of two different PGPR, allowed us to better understand their different effects on tomato plant, also extending the results from *in-vitro* assay. Indeed, this study highlight the importance of genome analysis as predictor of PGPR behavior on plant growth. We believe that the holistic comparative discussion, could enhance the overview of the issue about PGPR mode of actions on their eukaryotic host, cultured under greenhouse condition. Further studies are firmly necessary to establish the usefulness of molecular mechanism in less controlled environment.

## Data Availability Statement

The datasets presented in this study can be found in online repositories. The names of the repository/repositories and accession number(s) can be found at: NCBI SRA BioProject PRJNA699239: UC4115.

## Author Contributions

MG, EP, and MT: conceptualization. EP, MT, PC, and VT: methodology. MG, EF, and AF: formal analysis and writing— original draft. MG, EF, and EP: investigation. MG, AF, EF, EP, and VT: writing—review and editing. EP and MT: supervision. All authors contributed to the article and approved the submitted version.

## Conflict of Interest

The authors declare that the research was conducted in the absence of any commercial or financial relationships that could be construed as a potential conflict of interest.
