# Supplementary material for: Integrated Genomic and Greenhouse Assessment of a Novel Plant Growth-Promoting Rhizobacterium for Tomato Plant
Source: Front Plant Sci. 2021 Mar 30;12:660620. doi: 10.3389/fpls.2021.660620 (PMC8042378; doi:10.3389/fpls.2021.660620)
Supplement: Supplementary file 1 [file Table_1.DOCX]

**Supplementary**

**Table S1.** Conventional and organic peat composition

| **Peat** | **Mixture** | **Structure (mm)** | **pH value** | **Ferilizer (kg/cbm)** | **Nutrients (mg/l)** | | | | |
| --- | --- | --- | --- | --- | --- | --- | --- | --- | --- |
|  |  |  |  | **PG-Mix 14-10-18+micro** | **Organic N** | **NH_4_/NO_3_** | **P_2_O_5_** | **K_2_O** | **Mg** |
| Conventional | white peat and frozen through black peat | Extra fine (0 – 5) | 6.0 | 1,2 | - | 100/68 | 120 | 220 | 100 |
| Organic | white peat, frozen through black peat and TerrAktiv® | Extra fine (0 – 5) | 5.5 | - | 400 | 80 -120 | 150-250 | 350-500 | 100-200 |

**Table S2.** Genes related to PGP traits

| **Trait** | **PGfams ID** | **Gene annotation** | **KO/**  **Gene_ID** | **KEGG_**  **GENE_**  **NAME** | **UC**  **4115** | **Sp7** |
| --- | --- | --- | --- | --- | --- | --- |
| Nitrogenase  complex | PGF_00025954 | nitrogenase iron protein | K02588 | nifH | * | * |
|  | PGF_00025951 | nitrogenase molybdenum-iron protein alpha chain | K02586 | nifD | * | * |
|  | PGF_00025953 | nitrogenase molybdenum-iron protein beta chain | K02591 | nifK | * | * |
|  | PGF_00120348 | putative nitrogen fixation protein FixT | K02593 | nifT | * | * |
|  | PGF_00025669 | NifY protein | - | nifY | * | * |
|  | PGF_00025964 | nitrogenase MoFe cofactor biosynthesis protein NifE | K02587 | nifE | * | * |
|  | PGF_00945843 | nitrogenase iron-molybdenum-cofactor biosynthesis protein NifN | K02592 | nifN | * | * |
|  | PGF_00025961/  PGF_00025962 | nitrogen fixation protein NifX | K02596 | nifX | * | * |
|  | PGF_00420050 | cysteine desulfurase NifS | K04487 | nifS | * | * |
|  | PGF_00072326 | homocitrate synthase | K02594 | nifV | * | * |
|  | PGF_01958698 | putative NifW protein | K02595 | nifW | * | * |
|  | PGF_00025671 | putative NifZ protein | K02597 | nifZ | * | * |
|  | PGF_01971790 | nitrogen fixation protein NifM | K03769 | nifM | * |  |
|  | PGF_00071382 | flavodoxin FldA | K03839 | nifF | * |  |
|  | PGF_00029105 | nitrogen fixation negative regulator NifL /  PAS-PAC protein | K23916 | nifL | * |  |
|  | PGF_03973235 | Nif-specific regulatory protein | K02584 | nifA | * | * |
|  | PGF_06674514 | nitrogenase cofactor biosynthesis protein NifB | K02585 | nifB | * | * |
|  | PGF_00025976 | nitrogen fixation protein NifQ | K15790 | nifQ | * | * |
|  | PGF_00046007 | putative pyruvate:ferredoxin (flavodoxin) oxidoreductase | K03737 | nifJ | * |  |
|  | PGF_00015530 | Iron-sulfur cluster assembly scaffold protein | K04488 | nifU | * | * |
|  | PGF_00401722 | Nitrogenase-associated protein NifO | - | nifO | * | * |
| Nitrogenase  transport | PGF_00424131 | Na(+)-translocating NADH-quinone reductase subunit E | K03617 | rnfA | * |  |
|  | PGF_10542033 | electron transport complex protein RnfB; Required for nitrogen fixation. | K03616 | rnfB | * |  |
|  | PGF_04400591 | electron transport complex protein rnfC | K03615 | rnfC | * |  |
|  | PGF_01678333 | electron transport complex protein RnfD; Required for nitrogen fixation. | K03614 | rnfD | * |  |
|  | PGF_01484108 | NADH-ubiquinone oxidoreductase | K03613 | rnfE | * |  |
|  | PGF_00424141 | electron transport complex protein RnfG | K03612 | rnfG | * |  |
| Electron  transport | PGF_05015473 | Ferredoxin-like protein FixX/4Fe-4S ferredoxin | K03855 | FixX |  | * |
|  | PGF_03136394 | Electron transfer flavoprotein-quinone oxidoreductase FixC | K00313 | FixC |  | * |
|  | PGF_03134445 | Electron transfer flavoprotein, alpha subunit FixB | K03522 | FixB |  | * |
|  | PGF_03098759 | Electron transfer flavoprotein, beta subunit FixA | K03521 | FixA |  | * |
|  | PGF_00849787 | two-component system response regulator | K14987 | FixJ |  | * |
| Gluconic  acid | PGF_04577966 | quinoprotein glucose dehydrogenase/glucose dehydrogenase, PQQ-dependent | K00117 | gcd | * |  |
|  | PGF_01393943 | pyrroloquinoline-quinone synthase C | K06137 | pqqC | * | * |
|  | PGF_01084777 | coenzyme PQQ synthesis protein F | - | pqqF | * |  |
|  | PGF_00418484 | Coenzyme PQQ synthesis protein B | K06136 | pqqB | * | * |
|  | PGF_03579782 | Coenzyme PQQ synthesis protein D | K06138 | pqqD | * |  |
|  | PGF_00418486 | Coenzyme PQQ synthesis protein E | K06139 | pqqE | * | * |
| Phosphonate  transporter | PGF_12684827 | phosphonate ABC transporter, permease protein | K02042 | phnE1 | * |  |
|  | PGF_00033841 | phosphonate ABC transporter, permease protein | K02042 | phnE2 | * |  |
|  | PGF_00033852 | phosphate-binding protein of phosphonate ABC transporter | K02044 | phnD | * |  |
|  | PGF_00033832 | phosphonate transport system ATP-binding protein | K02041 | PhnC | * |  |
| Phosphate  transporter | PGF_07668761 | phosphate transport system substrate-binding protein | K02040 | Pst S | * | * |
|  | PGF_01072302 | phosphate transport system permease protein | K02038 | Pst A | * | * |
|  | PGF_02405545 | phosphate transport system permease protein | K02037 | Pst C | * | * |
|  | PGF_06213055 | phosphate transport system ATP-binding protein | K02036 | Pst B | * | * |
| Indole-3-acetic acid  (IAA) biosynthesis | PGF_05599542 | indole-3-pyruvate decarboxylase | K04103 | ipdC | * | * |
|  | PGF_00049805 | Amidase | K01426 | - | * |  |
|  | PGF_07597988 | aldehyde dehydrogenase (NAD+) (EC 1.2.1.3) |  | aldh | * | * |
|  | PGF_00418275 | nitrile hydratase, alpha subunit | K01721 | nthA | * | * |
|  | PGF_00418276 | nitrile hydratase, beta subunit | K20807 | nthB | * | * |
|  | PGF_03811905/  PGF_02254418 | histidinol-phosphate aminotransferase | K00817 | hisC | * | * |
| Siderophore  production | PGF_00015658/  PGF_00015659 | isochorismate/ Apo-aryl carrier protein | K01252 | entB, dhbB, vibB, mxcF | * | * |
|  | PGF_00025850/  PGF_07637567 | 2,3-dihydroxybenzoate-AMP ligase | K02363 | entE, dhbE, vibE, mxcE | * | * |
|  | PGF_05075091 | enterobactin synthetase component F | K02364 | entF | * | * |
|  | PGF_00424602 | Enterobactin exporter | K08225 | entS | * |  |
|  | PGF_00023831/  PGF_08225224 | 2,3-dihydro-2,3-dihydroxybenzoate dehydrogenase | K00216 | entA | * | * |
|  | PGF_00015696 | isochorismate synthase | K02361 | entC | * |  |
|  | PGF_00422373 | 4'-phosphopantetheinyl transferase EntD | K02362 | entD | * |  |
|  | PGF_00037591 | Proofreading thioesterase in enterobactin biosynthesis | K24147 | entH | * |  |
|  | PGF_07721642 | MbtH-like protein | K05375 | MbtH |  | * |
|  | PGF_00004447 | Ferric enterobactin-binding periplasmic protein | K23185 | FepB | * |  |
|  | PGF_00004441 | Ferric enterobactin transport system permease protein | K23186 | FepD | * |  |
|  | PGF_00004444 | Ferric enterobactin transport system permease protein | K23187 | FepG | * |  |
|  | PGF_00004439 | Ferric enterobactin transport ATP-binding protein | K23188 | FepC | * |  |
|  | PGF_00424600/  PGF_08225224 | Enterobactin esterase | K07214 | Fes | * | * |
|  | PGF_00057226 | TonB-dependent receptor; Outer membrane receptor for ferric enterobactin and colicins B, D | K19611 | FepA | * | * |
|  | PGF_00052044 | alternative sigma factor | - | PvdS |  | * |
|  | PGF_00045754 | PvdE, pyoverdine ABC export system, fused ATPase and permease components | K06160 | PvdE |  | * |
| 4-hydroxybenzoate  Production | PGF_00417843 | chorismate-pyruvate lyase | K03181 | ubiC | * |  |
| GABA | PGF_07204877 | succinate-semialdehyde dehydrogenase | K00135 | gabD | * | * |
|  | PGF_04337880 | 4-aminobutyrate aminotransferase | K07250 | gabT | * | * |
| Phenazine  biosynthesis | PGF_10329977 | Phenazine biosynthesis | - | phzF | * | * |
